# Comparison of dietary intakes of Canadian Armed Forces personnel consuming field rations in acute hot, cold, and temperate conditions with standardized infantry activities

**DOI:** 10.1186/s40779-019-0216-7

**Published:** 2019-08-16

**Authors:** Mavra Ahmed, Iva Mandic, Wendy Lou, Len Goodman, Ira Jacobs, Mary R. L’Abbé

**Affiliations:** 1 Department of Nutritional Sciences, Faculty of Medicine, University of Toronto, Medical Science Building, 1 King’s College Circle, Room 5368, Toronto, Ontario M5S 1A8 Canada; 2 Faculty of Kinesiology and Physical Education, University of Toronto, Toronto, M5S 2W6 Canada; 3 Dalla Lana School of Public Health, University of Toronto, Toronto, M5T 3M2 Canada; 4 Defence Research and Development Canada – Toronto Research Centre, Toronto, M3K 2C9 Canada

**Keywords:** Dietary assessment, Nutrient intakes, Military personnel, Physical activity, Temperature extremes, Field rations

## Abstract

**Background:**

Dietary Reference Intakes are used to guide the energy intake of the Canadian Armed Forces (CAF) field rations provided to military personnel deployed for training or operations. However, the high energy expenditures likely to occur under harsh environmental/metabolically challenging deployment conditions may not be adequately considered. This study examined the *Ad libitum *energy and nutrient intakes of CAF personnel (*n =* 18) consuming field rations in a resting thermoneutral environment and during a day of standardized strenuous infantry activities at varying environmental temperatures.

**Methods:**

Dietary intake was assessed using a measured food intake/food waste method during the experimental treatment and for 6 h after treatment. Four treatments were administered in a randomized counterbalanced design: exercise (as standardized infantry activities) in the heat (30 °C), exercise in the cold (− 10 °C), exercise in temperate thermoneutral (21 °C) air temperatures and a resting (sedentary) trial (21 °C).

**Results:**

The average *Ad libitum* consumption of field rations was 70% of the provided total energy (2776 ± 99 kcal/8 h) during all treatments. Even with an acute challenge of increased energy expenditure and temperature stress in the simulated field conditions, participants’ energy intakes (1985 ± 747 kcal/8 h) under hot, cold and temperate treatments did not differ from energy intake during the sedentary condition (1920 ± 640 kcal/8 h). Participants’ energy intakes (1009 ± 527 kcal/6 h) did not increase during the 6 h posttreatment period when the stresses of the strenuous physical activities and the harsh environmental temperatures had subsided.

**Conclusion:**

These results should be considered when planning the provision of field rations for CAF personnel expected to be engaged in strenuous physical activities with prolonged exposure to temperature extremes.

**Electronic supplementary material:**

The online version of this article (10.1186/s40779-019-0216-7) contains supplementary material, which is available to authorized users.

## Background

Military personnel frequently encounter metabolically demanding operations under conditions of environmental extremes (e.g., desert, arctic) [1–3]. The associated daily energy requirements have been reported to range from sedentary levels to as high as 10,000 kcal (kcal) [1, 2, 4]. The success of operations may in part be determined by the ability to adapt to extreme environmental conditions, including those adaptations that are dependent upon adequate nutrition [2, 5, 6].

Previous studies on the Canadian Armed Forces (CAF) and other military personnel have reported energy requirements in temperate/hot environmental extremes (10 °C to > 25 °C, depending upon humidity) to range from 3,300 kcal*/*d to 6,000 kcal/d [4, 7–12]. Similarly, energy requirements in cold environments (temperature < 10 °C, including arctic conditions) are reported to range from ~ 4,000 kcal/d to > 6,000 kcal/d [9, 12–16], but they can increase significantly due to physiological thermoregulatory responses (e.g., shivering); however, most of the energy expenditure in cold environments is attributed to heavier clothing and difficult terrain [2, 9, 13, 16, 17]. Although energy expenditures of CAF military units in the field are comparable to those of other military populations, information on adequate nutrient requirements from other military populations may not be generalizable to the CAF military personnel due to differences in demographics, training requirements, standard operating procedures, trades/types of duties, and differences in the components of the field rations.

Operating in ambient temperature extremes has also been associated with changes in physiological and nutrient requirements [2, 6]. For example, military personnel lose a significant amount of sodium through sweat and are also at risk of hypohydration as a result of operating in a hot environment, thus requiring adequate sodium and water intakes [18–20]. Although the energy requirements of personnel are largely dependent on activity levels and other factors, loss of appetite has been demonstrated as a consequence of working in a hot environment, which increases the risk of suboptimal energy intake in relation to energy requirements [12, 21, 22]. Lower energy intake has also been observed in sedentary conditions with ambient temperature extremes [23]. In contrast, increases in appetite and energy intake have been demonstrated in cold environments (both with and without strenuous physical activity) [12, 21, 23]. Both dehydration and hypohydration are also concerns in the cold due to exercise-induced respiratory water losses, sweating and decreased thirst sensations [24–26]. Overall, it is well established that maintaining adequate nutritional and hydration status is integral to individual operational readiness [2]. Additionally, research also indicates that there might be altered requirements for micronutrients under temperature extremes as a result of environmental stress impacting intestinal absorption and/or increased utilization of some of these nutrients [16, 27].

Military field rations are the principal source of nutrients provided to CAF personnel during the initial stages of field operations, and they may be consumed for weeks during operations in many environments until military field kitchens are established or fresh food from local sources can be obtained [28]. These rations are not meant to be used exclusively beyond 30 consecutive days, and where possible, military personnel are supplemented with fresh foods after 14 days of exclusive subsistence on field rations [29, 30]. However, the provision of fresh foods is not always possible after 14 days [9], and missions may be longer than intended; thus, some CAF personnel may end up consuming field rations for longer than 30 days [9]. The current feeding practices for CAF personnel during deployments/training adhere to the CAF Food Services standards, which follow the Dietary Reference Intake (DRI) recommendations and Canada’s Food Guide (CFG) [9, 30]. The nutritional recommendations (specifically the type of food provided, e.g., fresh foods or field rations) for CAF personnel in the garrison, in the field and/or in combat/training depend on the logistical and tactical components of the deployment [9, 28]. In addition to the standard food allowances, supplemental food (or incremental allowances, IAs) may be provided in the field under conditions when it is suspected that standard allowances may be insufficient to fuel the energy demands of metabolically challenging operations under environmental extremes [9, 30].

Although high energy expenditures are expected during field operations, particularly those involving ambient temperature extremes, the IAs are not based on empirical evidence of the increased energy demands under such conditions [9], even though such arduous conditions have been repeatedly reported to be associated with a gap between the amounts of food provided and the amount that is actually consumed [2, 31]. The gap is likely the end result of a combination of factors that include appetite suppression, palatability/variety of rations provided, methods and time required for food preparation and consumption, and load being carried [9, 32]. As repeatedly demonstrated in the literature, these factors contribute to voluntary anorexia, which is defined as the failure to consume foods that are offered or readily available under situations of extreme stress [4, 12]. This voluntary anorexia can have negative psychological and physiological consequences, thereby impacting operational readiness and performance [2, 33, 34].

It is likely that military personnel may compensate for voluntary anorexia by increasing their energy intake when time permits or when the situations of stress have ended. However, the strenuous physical activity and the environmental temperature at which the exercise is performed may also influence the energy intake from the meals consumed postoperation [35, 36]. The extent to which CAF military personnel may compensate for this voluntary anorexia during operations in harsh environmental conditions by subsequently consuming additional energy after operations have concluded and/or when they have time to eat is unknown.

There is a paucity of research in the recent literature about the energy intake of CAF personnel in relation to energy needs as a result of strenuous physical activities in harsh environmental temperatures. Most of the studies about CAF personnel do not include accurate assessments of energy and nutrient intakes or accurate measures of energy expenditure [13, 33] nor have they investigated the interactions of energy content of rations with ambient temperature extremes [1, 13, 33]. Furthermore, several of these studies are dated, and since then, there have been changes in the demographics of CAF personnel, advances in CAF tactics/technologies or operational duties and changes in nutritional intakes and modified perceptions of ‘healthy’ foods [37].

Using the current gold standard in dietary assessment methodology, the measured food intake/waste collection method [38], the primary objectives of this study were to 1) measure energy and nutrient intakes of CAF personnel consuming field rations *Ad libitum* in an experimental laboratory setting using a varying temperature and humidity controlled chamber during an 8 h day while performing standardized physically demanding infantry activities and 2) measure energy and nutrient intakes from subsequent *Ad libitum* ration consumption after the experimental trials to determine the degree of energy compensation following the different treatments.

## Methods

### Study participants

A total of 27 participants initially volunteered for the study. The volunteers were not selected to be representative of the entire CAF population but rather were a convenience sample of the Regular Force and Class A Reservists. Two participants never started the protocol, and an additional 7 participants dropped out due to scheduling difficulties and/or noncompliance with the demanding nature of the study protocol. The remaining 18 CAF personnel completed all 4 treatment phases. All participants provided written informed consent, and this study protocol was approved by Defence Research & Development Canada (DRDC) (#2013–075) and University of Toronto (#29914) Institutional Human Research Ethics Boards.

### Experimental design and inclusion criteria

**Fig. 1:**
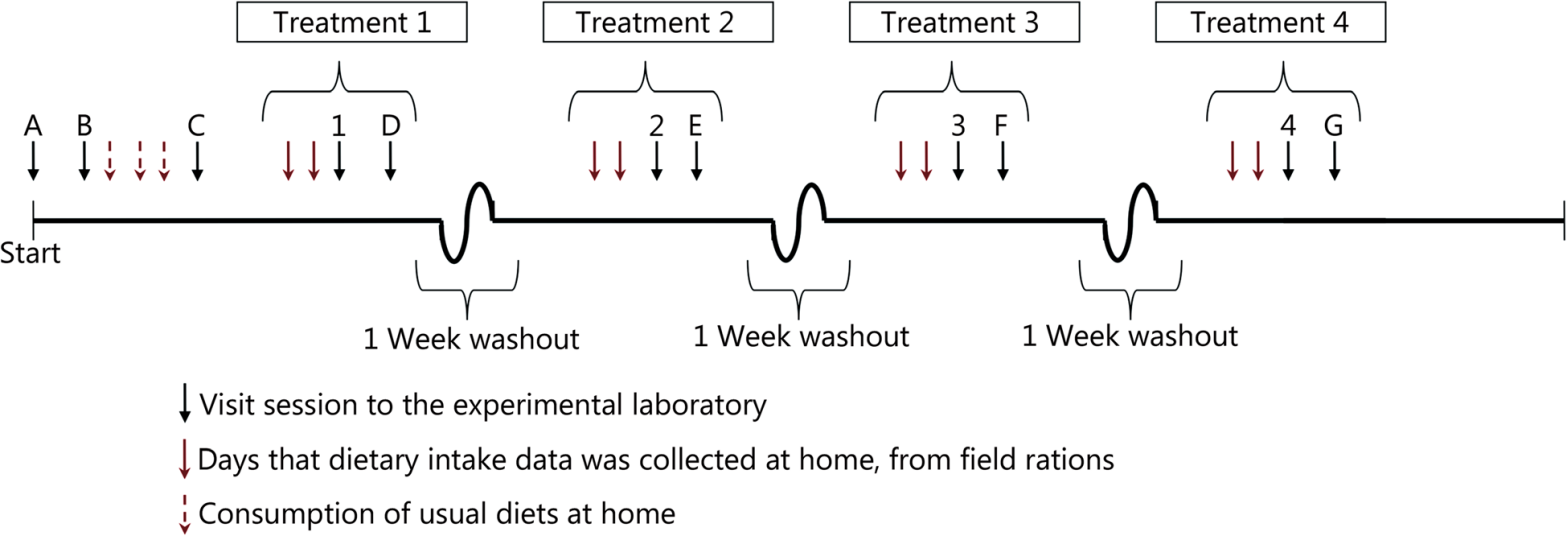
Experimental design of the laboratory simulation trial with standardized strenuous infantry activities conducted in an environmentally controlled chamber. A randomized crossover experimental design was implemented in which treatments were hot, cold, temperate (with strenuous physical activities) and sedentary. Treatments 1–4 refer to the 8 h-long experimental treatments in the temperature- and humidity-controlled environmental chamber. The red dashed arrows represent the consumption of usual diets at home (this information is not presented in this manuscript). The solid red arrows represent days that participants consumed field rations at home. The following were collected and/or measured at the study visit: session A (informed consent), B (sociodemographic/anthropometric information, maximal oxygen consumption, PARQ^+^), C (body composition and urinary/blood biomarkers) and D, E, F, G (urinary and blood biomarkers and completion of questionnaires)

The study was conducted with a randomized crossover design in which each participant attended the laboratory on six separate occasions. The two initial visits consisted of the informed consent process, completion of the Physical Activity Readiness Questionnaire-Plus (PAR-Q^+^), collection of demographic data and anthropometric measurements, measurement of maximal aerobic power (*V̇O*_*2peak*_ measured using indirect calorimetry during an incremental treadmill exercise test to exhaustion [39]) and percent body fat (measured via air-displacement plethysmography [BOD POD™, COSMED, Rome, Italy]). The PAR-Q^+^ and the measurement of maximal aerobic power were administered prior to enrollment in the study and used as inclusion criteria to determine if the participants were fit for exercise without requiring secondary clearance by the Canadian Forces Environmental Medical Establishment (DRDC). The subsequent visits consisted of four experimental trials conducted in an environmental chamber (temperature and humidity controlled). These trials were administered in a randomized counterbalanced design: exercise (as standardized infantry activities) in the heat (30 °C), exercise in the cold (− 10 °C), exercise in temperate thermoneutral air temperatures (21 °C) and a resting (sedentary) trial (21 °C, Fig. 1).

### Demographic and physical characteristics

Participants were asked to complete a questionnaire on demographics that included information on ethnicity and education level. Anthropometric measurements included height, weight, and body fat percentage. Body weight and height were measured without shoes, with light clothing using standard, calibrated equipment (height and weight scales – HealthOMeter Continental Scale Corp., Bridgeview IL, USA). Body composition (including percent body fat) was assessed using air-displacement plethysmography (BOD POD™). Body mass index (BMI) was calculated as the body weight (kg) divided by the height (m) squared.

Of the 18 CAF participants, 14 were male and 12 were Caucasian. The mean age was 34 ± 11 years, with a mean weight of 79 ± 13 kg, a mean BMI of 26 ± 4 kg/m^2^ and a mean body fat percentage of 23% ± 8%. The majority of the participants had a university degree (61%).

### Dietary intake assessment

The primary objective of this study was to assess the energy and nutrient intakes consumed *Ad libitum* from the field rations during the experimental trial sessions (8 h) and during the subsequent postexperimental sessions (6 h). Complete dietary intake data were presented for both the 8 h experimental trial sessions and for the postexperimental sessions (6 h) (as participants were asked to fast prior to coming back the next day for a blood test). Energy and macronutrient intakes were expressed per 24 h period to compare them to the 24 h energy expenditures. The present manuscript has a companion study that provides detailed methods and results on energy expenditure and appetite-related hormones [40].

For each experimental trial, participants were able to consume food *Ad libitum* from three self-selected standard military ration packs. Although the menu items differed between participants, each participant consumed the exact same ration packs for their 4 treatment visits.

Canadian field rations are shelf-stable (3 years), precooked, ready-to-eat foods and beverages [9, 28]. Field rations offer common foods based on Canadian preferences and eating patterns and contain several meatless options [30]. All rations were procured and used within their stable self-life and stored according to specified instructions by the CAF Directorate of Food Services [28]. The rations contain prepackaged, pre-labeled food and beverage items (e.g., sliced apples, bread, coffee, breakfast sausages, etc.) and met the AMDRs for macronutrients (Table 1).

**Table 1:** Energy and macronutrients available and consumed from self-selected individual meal packs or field rations provided to participants during the 8 h experimental treatments and during the postexperimental treatments (total of 24 h).[*Mean ± SD*]

Individual meal pack^1^	Energy (kcal)	Carbohydrates (g)	Total fat (g)	Protein (g)
Breakfast	1344 ± 104	221 ± 31	41 ± 11	31 ± 5
Lunch	1432 ± 78	224 ± 22	41 ± 10	43 ± 7
Dinner/Supper	1317 ± 81	200 ± 21(61%)^5^	39 ± 8 (26%)^5^	42 ± 10 (13%)^5^
LMC	1125 ± 172	203 ± 23 (72%)^5^	25 ± 3 (20%)^5^	35 ± 1 (12%)^5^
DRI (g)/AMDR (%)^2^(/d)	2800–3200	338–488 (45–65%)	67–117 (20–35%)	75–262 (10–35%)
Total available^3^/ 8 h (% of total energy)	2776 ± 99	445 ± 25 (64%)	82 ± 10 (27%)	74 ± 9 (11%)
Total available^3^/ 24 h (% of total energy)	4093 ± 97	645 ± 26 (63%)	121 ± 9 (26%)	116 ± 9 (11%)
Average consumed^4^/ 8 h	1970 ± 718	295 ± 119 (59%)	61 ± 23 (28%)	63 ± 23 (13%)
Average consumed^4^/ 24 h	2979 ± 918	443 ± 153 (59%)	92 ± 29 (28%)	99 ± 26 (13%)

^1^Individual Meal Packs or field rations consisted of a choice of a variety of foods and beverages from 18 menu items (6 breakfast, 6 lunch and 6 dinner/supper), which participants (*n =* 18) preselected for their experimental treatments. Once selected, each participant consumed *Ad libitum* from the exact same menu items during each of the experimental treatments (sedentary, 21 °C), cold (− 10 °C), hot (30 °C) and temperate (21 °C); humidity (~ 30% in the sedentary, hot and temperate treatment and 50% in the cold treatments). The 24 h data included at home 6 h posttreatment meals. ^2^Dietary Reference Intakes (DRI)/ Average Macronutrient Distribution Ranges (AMDR) [45] (for a moderately active to highly active 70 kg male); Carbohydrates, fat and protein AMDR values were based on an average intake of 3000 kcal/d. ^3^The total available/d corresponds to the total amount of nutrients available for consumption if the three ration packs (breakfast, lunch and dinner) were consumed in their entirety.^4^Consumed indicates the average amount of energy and macronutrients eaten during the 8 h experimental treatments (in the environmentally controlled chamber) and during the 24 h (including the at home 6 h posttreatment) (as measured using the measured food intake/food waste collection method). ^5^ The percentages indicate the energy contribution of the macronutrients where the data were calculated using the energy contribution of 4 kcal/g for carbohydrates and protein and 9 kcal/g for fat. AMDR. Average Macronutrient Distribution Range; LMC. Light Meal Combat

**Fig. 2:**
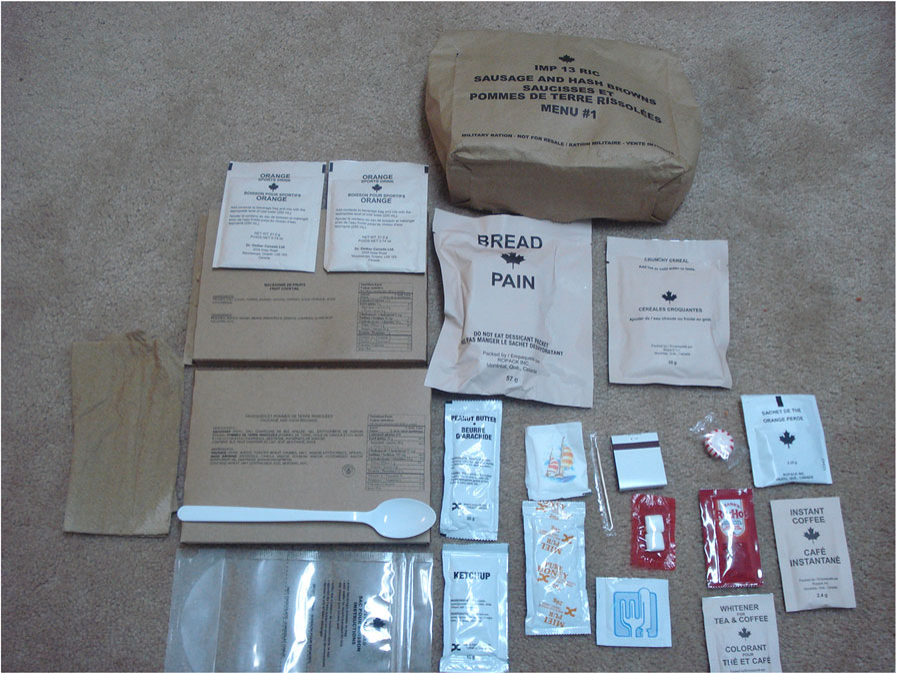
An example of an individual meal pack (IMP). IMPs typically contain an entrée, fruit, cereal (breakfast), bread option, beverage options (e.g., hot beverages/sports drink crystals), spreads/condiments (e.g., jam/peanut butter, hot sauce, sugar), candy/chewing gum, salt/pepper, utensils, beverage pouch, matches, Kim towel, toothpick, and a moist towelette

Each military ration is composed of a main entrée (e.g., lasagna), dessert (e.g., blueberry apple sauce), sport drink crystals (e.g., grape, orange), bread/tortillas, condiments (jam/peanut butter/honey), powdered mixes for beverages (hot chocolate, instant coffee), seasonings (e.g., salt/pepper), and confectionaries (chocolate bars, candy, gum) (Fig. 2). The light meal combat ration pack consists of a snack (e.g., beef jerky, fruit/energy bars) and beverage items (sport drink crystals, hot chocolate). The foods and beverages within these rations are of precisely known quantity and nutrient composition. Although participants could only consume the beverages (e.g., sports drink, coffee, tea, vanilla cappuccino) provided within the rations, they were able to have water *Ad libitum* during the experimental treatments and at home posttreatment*.* Participants also self-selected a dinner/supper ration pack and/or light meal combat ration pack to consume *Ad libitum* later that day under thermoneutral conditions (e.g., at home).

Dietary intake was assessed using the measured food intake/waste method (considered the current gold standard for dietary assessment [38]) on each of the two days prior to the experimental treatments and on each of the four experimental treatment days. Study coordinators were in the chamber with the participant for all of their visits and documented the consumption of foods and/or beverages (including water). All unconsumed and/or partially consumed food and beverage items were weighed and recorded by study coordinators to the nearest gram (g) or milliliter (ml), using a standard food scale (PrepTech, PT-800, Newport Beach, CA). The consumption of field rations from dinner ration packs was assessed using the measured food intake/waste method, as participants were asked to bring back any leftover/partially consumed foods the day after the experimental visit. The measured food intake/food waste was calculated from the amount unconsumed subtracted from the known quantity of each menu item selected [41].

Food intake was entered by two trained coders using a nutrient software program ESHA© Food Processor SQL (version 10.13.1, 2013, ESHA© Research, Salem, OR, US), which was preloaded with nutritional information for all the food and beverage components found within the field rations, and the food intake data were double-checked and analyzed by the study investigator. Water intake was calculated from water consumed as plain water, water mixed with a beverage/sports drink or water mixed with food. The nutrient values of the foods and beverages in the combat/field rations were provided by CAF Directorate of Food Services based on manufacturer specifications and/or by chemical analyses and were available as a Nutrition Facts label on the ration components. For nutrients that were missing (B vitamins, potassium, magnesium, phosphorus, zinc), values were taken from similar foods in the Canadian Nutrient File 2013 [42] as part of the ESHA©(Elizabeth Stewart Hands and Associates, ESHA©) Food Processor Nutrition Analysis software (version 10.13.1, 2013, ESHA Research, Salem, OR, USA).

### Experimental trials

Each participant completed the four experimental treatments individually with a 1-week washout between treatments. Participants were asked to refrain from alcohol consumption and vigorous physical activity in the 48 h preceding the experimental treatments. Participants self-selected three standard military ration packs (of the 18 available ration menu items [6 breakfast, 6 lunch and 6 dinner]) per experimental trial. The ration choices that each study participant selected were then provided for *Ad libitum* consumption at each of the four experimental trial treatment phases.

For each of the 4 experimental trials, participants arrived at the laboratory at 7:00 A.M. (Military Time 0700 h) in a fasted state. Prior to arriving to the laboratory, participants swallowed a telemetric core temperature capsule with 250 ml of water at home to measure and monitor their core temperature using the Equivital™ receiver (Hidalgo, Cambridge, United Kingdom). On arrival, participants were weighed and had their resting blood pressure measured using an automated blood pressure cuff (Omron Health Care, Kyoto, Japan). According to the inclusion criteria, on the day of the experimental visit, as required by the Canadian Forces Environmental Medical Establishment (DRDC) for the safety of the participants, resting blood pressure was measured to ensure that participants’ blood pressure was not above 140/90 before the start of the trial and the temperature was monitored to ensure that their core temperature at any point in the trial did not exceed 39.5 °C. An initial blood sample (10 ml) was taken from an antecubital vein (erythrocytes and plasma were separated within 1 h of collection), and then participants were provided with the breakfast meal from their self-selected ration packs. Blood samples were taken for the assessment of plasma concentrations of nutritional biomarkers (results provided in this manuscript) and appetite hormones (leptin, acylated ghrelin, GLP-1 and PPY) (for detailed analyses on appetite hormones, see Mandic et al. [40]). The visual analogue scales for appetite (VASA) [43] were administered throughout the trial (for detailed analyses, see Mandic et al. [40]). Standardized food satisfaction surveys were also administered to assess the field rations selected (Additional file 1: Table S1). Participants were instructed to wear temperature-appropriate standard military clothing for the environmental treatments they were to complete. The clothing included military fatigues, a tactical vest, a fragmentation protection vest, combat boots and a helmet (weight of clothing was similar between participants [40]). Energy expenditure was measured for 4 hrs continuously (during the activity sections of the experimental visit) using a portable metabolic measurement system (Metamax 3B, CORTEX Biophysik GmbH, Leipzig, Germany), and heart rate was measured using a heart rate telemetry chest strap (Polar, Kempele, Finland). For detailed analyses of energy expenditure, see Mandic et al. [40].

Participants were provided with detailed instructions on how to collect their urine for 24 h (including 8 h in the chamber and at home posttreatment). Participants were provided with a plastic container to collect their urine and a leak-proof bag in which to store their urine container. Participants were instructed to discard the first urine sample of the day and to collect all subsequent urine for the next 24 h, including the first urine sample on the following day. Both the urine and plasma samples for the assessment of nutritional biomarkers were shipped to a third-party blood chemistry laboratory (Lifelabs, Toronto, ON, Canada) for processing. Creatinine excretion was used to assess the adequacy of the urine collections using creatinine excretion standards (< 8.8 mmol/d for males and < 4.5 mmol/d for females) [44].

Participants entered the environmental chamber in a randomized order, which was maintained under the following conditions: hot (30 ± 0.2 °C, humidity 31% ± 1%), cold (− 10 ± 0.4 °C, humidity 56% ± 3%), temperate thermoneutral (21 ± 0.2 °C, humidity 32% ± 4%) and a resting (sedentary) trial (21 ± 0.3 °C, humidity 29% ± 2%, Fig. 1). Following entry into the chamber, participants either rested for 8 h (sedentary) or performed 2 h activity circuits composed of standardized typical military tasks (or infantry activities, covering a range of light, moderate and heavy workloads), followed by a 2 h rest period (for 2 circuits/d) for a total 4 h of physical activity with 4 h of rest in between each circuit, during which they could consume foods and beverages *Ad libitum* from the self-selected ration packs. The standardized infantry activities completed during the circuits included both aerobic (e.g., treadmill walking at various speeds, inclines, and while carrying different loads) and anaerobic (e.g., simulating stretcher carry, lifting ammo cans and jerry cans, dragging an 85 kg dummy, etc.) activities [40]. Examples of sedentary activities were sitting, kneeling, and assuming the drop and fire position. Study coordinators prepared the foods and beverages from the ration packs prior to the 2 h rest periods based on the participants’ requests. The preparation of the food items typically required heating the food item with a flameless food heating sleeve (Truetech Inc. Riverhead, NY, US) and/or adding hot water (e.g., for items such as cereals, rice, couscous), which was done outside the environmental chamber. Study coordinators also prepared the sports drink and/or coffee-based beverages (preparation requires addition of hot or cold water) if participants requested these items while performing the physical activities or resting.

At the end of each experimental visit day, participants were provided with their selected dinner ration pack and/or light meal combat (LMC) ration pack to consume later that day (the rest of the day was an approximately 6 h postexperimental treatment since they were asked to fast for subsequent blood collection the day after their treatment visit day) under thermoneutral conditions (e.g., at home) and were instructed to refrain from consuming any foods or beverages (except water) not found within the field rations, including dietary supplements and performance enhancers. Participants could choose additional food in the form of a light meal combat ration pack (on top of the standard IMPs) if they wished (and they could consume these rations *Ad libitum*). On the next day, participants were instructed to bring back all unconsumed and/or partially consumed rations.

### Statistical analyses

The study design was a repeated-measures crossover design, and the sample size calculation was based on detecting a 10% difference in energy intake between treatments with 80% power at an alpha of 0.05. Nutrient data are presented as the mean ± standard deviation (SD) or as a percentage of total energy. Energy, macronutrient and micronutrient intakes were compared between the experimental treatments using a linear mixed model that accounts for repeated measures within participants with condition as fixed factors, visits as repeated effects and with post hoc pairwise comparisons using Bonferroni adjustment to determine statistical significance. Generalized linear mixed models with gamma distributions were used for nonnormally distributed data. Macronutrient intakes were assessed for adequacy in comparison with Dietary Reference Intake (DRI) recommendations for Average Macronutrient Distribution Ranges (AMDR) for carbohydrates, fat and protein for moderately active to highly active individuals [45]. Linear mixed models were used to compare differences in urinary and blood biomarkers between experimental conditions, and the urinary biomarkers were adjusted for total volume of urine. All data were analyzed using SPSS (version 24, 2016, IBM Corporation®, Armonk, NY, US). The *P*-value ≤0.05 was considered significant.

## Results

### Total energy and macronutrient intakes during the 8 h experimental trials and for 24 h across all experimental trials

There were no significant differences in energy or nutrient composition between the rations, and consistent energy was selected by all participants (Table 1). Participants consumed approximately 70% of the energy (average for the four experimental treatments) from the self-selected field rations during the 8 h treatment visit (Table 1). For all treatments, the average energy intake was 1970 ± 718 kcal/8 h with total energy from carbohydrates, fat and protein of 59, 28, and 13%, respectively, which were within the AMDR recommendations (Table 1). There were no significant differences in energy or macronutrient intake between the experimental treatments (*P >* 0.05, Table 2).

**Table 2:** Comparison of the effects of the different treatments on energy and nutrient intake by CAF personnel during the 8 h experimental treatment period. [*Mean ± SD*]

Nutrient intake per 8 h ^1^	Average for experimental treatments	Sedentary	Strenuous physical activity^3^			*P*-value
			Cold	Hot	Temperate	
Energy (kcal)	1970 ± 718	1920 ± 640	1897 ± 793	2005 ± 795	2055 ± 680	0.56
Carbohydrates (g)	295 ± 119	279 ± 105	286 ± 129	298 ± 128	315 ± 118	0.47
Total fat (g)	61 ± 23	62 ± 23	58 ± 25	63 ± 26	61 ± 20	0.44
Saturated fat (g)	22 ± 9	22 ± 9	22 ± 10	22 ± 11	21 ± 8	0.89
Protein (g)	63 ± 23	64 ± 20	62 ± 28	64 ± 25	64 ± 20	0.76
Fiber (g)	19 ± 9	18 ± 7	19 ± 10	20 ± 9	19 ± 8	0.77
Total sugar (g)	139 ± 58	130 ± 45	132 ± 59	147 ± 59	149 ± 67	0.34
Sodium (mg)	3183 ± 1183	3022 ± 1111	3353 ± 1276	3005 ± 1063	3350 ± 1313	0.25
Potassium (mg)	527 ± 218	531 ± 208	474 ± 220	532 ± 200	571 ± 248	0.24
Vitamin A (μg) (RE)	263 ± 228	320 ± 266	213 ± 183	248 ± 221	271 ± 243	0.18
Calcium (mg)	526 ± 257	435 ± 238^a^	519 ± 283^ab^	620 ± 275^b^	530 ± 216^ab^	0.017^*^
Magnesium (mg)	118 ± 48	104 ± 43^a^	103 ± 53^a^	133 ± 41^b^	131 ± 50^b^	0.004^*^
Iron (mg)	21 ± 12	14 ± 6	14 ± 8	15 ± 7	15 ± 6	0.64
Phosphorus (mg)	267 ± 126	272 ± 126	239 ± 117	267 ± 129	289 ± 138	0.28
Zinc (mg)	3.5 ± 1.9	3.3 ± 1.4	3.3 ± 2.3	3.8 ± 2.2	3.7 ± 1.5	0.51
Vitamin B_1_ (mg)	0.31 ± 0.20	0.25 ± 0.15	0.30 ± 0.21	0.33 ± 0.22	0.37 ± 0.20	0.10
Vitamin B_2_ (mg)	1.1 ± 3.5	0.23 ± 0.14	1.9 ± 4.8	1.1 ± 3.5	1.2 ± 3.5	0.25
Vitamin B_3_ (mg) (NE)	7.8 ± 4.9	8.3 ± 4.6	6.5 ± 4.7	7.7 ± 4.6	8.8 ± 5.7	0.11
Vitamin B_6_ (mg)	0.50 ± 0.38	0.59 ± 0.42	0.36 ± 0.27	0.56 ± 0.4	0.47 ± 0.38	0.19
Vitamin B_12_ (μg)	0.92 ± 1.11	0.97 ± 1.32	0.64 ± 0.85	0.99 ± 1.08	1.08 ± 1.19	0.23
Folate (μg) (DFE)	93 ± 72	96 ± 68	74 ± 72	95 ± 71	107 ± 77	0.24
Vitamin C (mg)^2^	128 ± 128	88 ± 86 ^a^	93 ± 123 ^a^	147 ± 112 ^b^	184 ± 164 ^b^	0.02^*^
Caffeine (mg)	67 ± 79	55 ± 43 ^a^	109 ± 100 ^b^	35 ± 48 ^a^	70 ± 95 ^ab^	0.005^*^
Water (ml)	2678 ± 1502	1280 ± 536 ^a^	1843 ± 1050 ^b^	4148 ± 1086 ^c^	3440 ± 1071 ^d^	< 0.001^*^

Study participants were Regular Force or Class A Reservists (*n =* 18). The data presented are for 8 h of nutrient intake by participants overall and for each of the four randomized experimental treatment groups [sedentary (21 °C), cold (− 10 °C), hot (30 °C) and temperate (21 °C); humidity (~ 30–50%)]. Energy and nutrient intake data were collected using the measured food intake/food waste method, where all consumed and/or non/partially consumed food and beverage items were weighed and recorded for each participant. ^1^Energy and nutrient intake data were examined by Linear Mixed Models with Bonferroni adjustment. The consumption of ^2^Vitamin C-fortified drink crystal packets (average count) was as follows: 0.9 (sedentary), 0.9 (cold), 2.4 (hot), and 2.6 (temperate). ^3^Strenuous physical activity consisted of standardized infantry activities. Data are presented as the means ± standard deviations (SDs). ^a,b^ Values with different superscripts indicate statistically significant differences (^*^P ≤ 0.05). RE. Retinol Equivalents; DFE. Dietary Folate Equivalents; NE. Niacin Equivalents

### Micronutrient and water intakes

Vitamin C was highest in the temperate treatment group compared to the level in the sedentary and cold treatment groups (*P* ≤ 0.05), but the level was not different than that associated with the hot treatment (Table 2). There were no significant differences between treatments for other micronutrients (including vitamin A and iron, *P* > 0.05), except calcium intake, which was significantly different between the sedentary and hot treatments (*P* ≤ 0.05); however, there were no differences when the sedentary treatment was compared to the cold or temperate treatments (*P* > 0.05). There were no significant differences in sodium intake between the experimental treatments (*P* > 0.05), averaging 3183 ± 1183 mg/8 h (mean ± SD) for all four treatments, although magnesium levels were higher in the hot and temperate treatment groups (*P* ≤ 0.05). Water intake was highest in the heat followed by the temperate, cold and sedentary treatments (*P* ≤ 0.05), while caffeine intake was highest in the cold treatment group (*P* ≤ 0.05).

### Total energy and macronutrient intakes postexperimental treatments

There were no differences in *Ad libitum* energy or macronutrient intake among experimental treatments from the evening meal/meals (time period averaged 6 h in the postexperimental treatments). Participants consumed an additional 1009 kcal (average for all treatments) in the evening from the self-selected rations (average content in a dinner ration pack was 1317 kcal, Tables 1 and 3). The total energy available to consume from three field rations (and/or LMC) during the 24 h was 4093 kcal, but participants consumed 2979 kcal/d (72.8%, Table 1). Overall, there were no differences in energy intake among the experimental treatments during the 8 h when the participants were in the environmentally controlled chamber or for the remaining 6 h during the at-home posttreatments (Table 3).

**Table 3:** Comparison of the effects of the different treatments on energy and nutrient intakes by CAF personnel from at-home consumption of dinner ration packs during the 6 h post treatment period. [*Mean ± SD*]

Nutrient intakes in postexperimental treatments (at home) ^1^	Averaged for postexperimental treatments	Post-sedentary (temperate)	Post- strenuous physical activity^2^			*P-*value*
			Post-cold	Post-hot	Post-temperate	
Energy (kcal)	1009 ± 527	1014 ± 701	968 ± 431	1140 ± 528	919 ± 358	0.62
Carbohydrates (g)	148 ± 86	145 ± 114	142 ± 67	170 ± 88	135 ± 54	0.60
Total fat (g)	31 ± 17	32 ± 20	30 ± 17	32 ± 20	28 ± 12	0.84
Saturated Fat (g)	12 ± 8	12 ± 9	11 ± 8	14 ± 9	11 ± 6	0.57
Protein (g)	36 ± 17	37 ± 21	33 ± 13	42 ± 17	30 ± 12	0.18
Fiber (g)	8 ± 4	8 ± 6	7 ± 3	9 ± 5	7 ± 4	0.40
Total sugar (g)	79 ± 52	81 ± 69	77 ± 43	85 ± 56	72 ± 39	0.87
Sodium (mg)	1297 ± 818	1331 ± 1088	1344 ± 732	1366 ± 770	1146 ± 671	0.77
Potassium (mg)	287 ± 196	304 ± 264	298 ± 168	313 ± 196	233 ± 142	0.53
Vitamin A (μg) (RE)	583 ± 639	552 ± 513	798 ± 959	529 ± 491	456 ± 456	0.40
Calcium (mg)	186 ± 129	186 ± 137	167 ± 112	222 ± 162	168 ± 98	0.35
Magnesium (mg)	65 ± 37	65 ± 34	64 ± 27	77 ± 53	54 ± 28	0.33
Iron (mg)	15 ± 10	15 ± 10	16 ± 8	15 ± 12	13 ± 10	0.80
Phosphorus (mg)	204 ± 96	226 ± 85	210 ± 82	214 ± 125	168 ± 82	0.27
Zinc (mg)	3 ± 2	3 ± 1.7	3 ± 1.7	3 ± 1.7	2.5 ± 1.4	0.59
Vitamin B_1_ (mg)	0.22 ± 0.15	0.25 ± 0.16	0.24 ± 0.14	0.20 ± 0.16	0.19 ± 0.16	0.49
Vitamin B_2_ (mg)	0.65 ± 2.5	1.14 ± 3.66	0.25 ± 0.11	0.21 ± 0.13	1.03 ± 3.58	0.58
Vitamin B_3_ (mg) (NE)	5.5 ± 3.6	5.8 ± 3	6.3 ± 3.7	5.2 ± 5	4.7 ± 3	0.55
Vitamin B_6_ (mg)	0.28 ± 0.24	0.30 ± 0.22	0.36 ± 0.33	0.26 ± 0.24	0.22 ± 0.14	0.35
Vitamin B_12_ (μg)	0.73 ± 0.99	1.02 ± 1.19	1.04 ± 1.11	0.31 ± 0.67	0.57 ± 0.83	0.05
Folate (μg) (DFE)	76 ± 65	74 ± 66	81 ± 49	81 ± 88	69 ± 51	0.84
Vitamin C (mg)	89 ± 94	90 ± 113	83 ± 81	98 ± 108	83 ± 78	0.91

Study participants were Regular Force or Class A Reservists (*n =* 18). The data presented are energy and nutrient intakes of participants overall and after each of the 8 h experimental treatments [sedentary (21 °C), cold (−10 °C), hot (30 °C) and temperate (21 °C); humidity (~ 30% in the sedentary, hot and temperate treatments and 50% in the cold treatment)]. Energy and nutrient intake data were collected using the measured food intake/food waste method, where all consumed and/or non/partially consumed food and beverage items from the field ration packs were weighed and recorded for each participant. ^1^Energy and nutrient intake data were examined by Linear Mixed Models with Bonferroni adjustment. ^2^Strenuous physical activity consisted of infantry activities during the 8 h experimental treatments. Data are presented as the means ± standard deviations (SDs). * *P*-value is the significance level for differences between the experimental treatments (postsedentary, postcold, posthot and posttemperate). Water intake was not measured during the postexperimental treatment period. RE. Retinol equivalents; DFE. Dietary folate equivalents; NE. Niacin equivalents

### Energy intake in comparison to energy expenditure (8 h and 24 h)

**Fig. 3:**
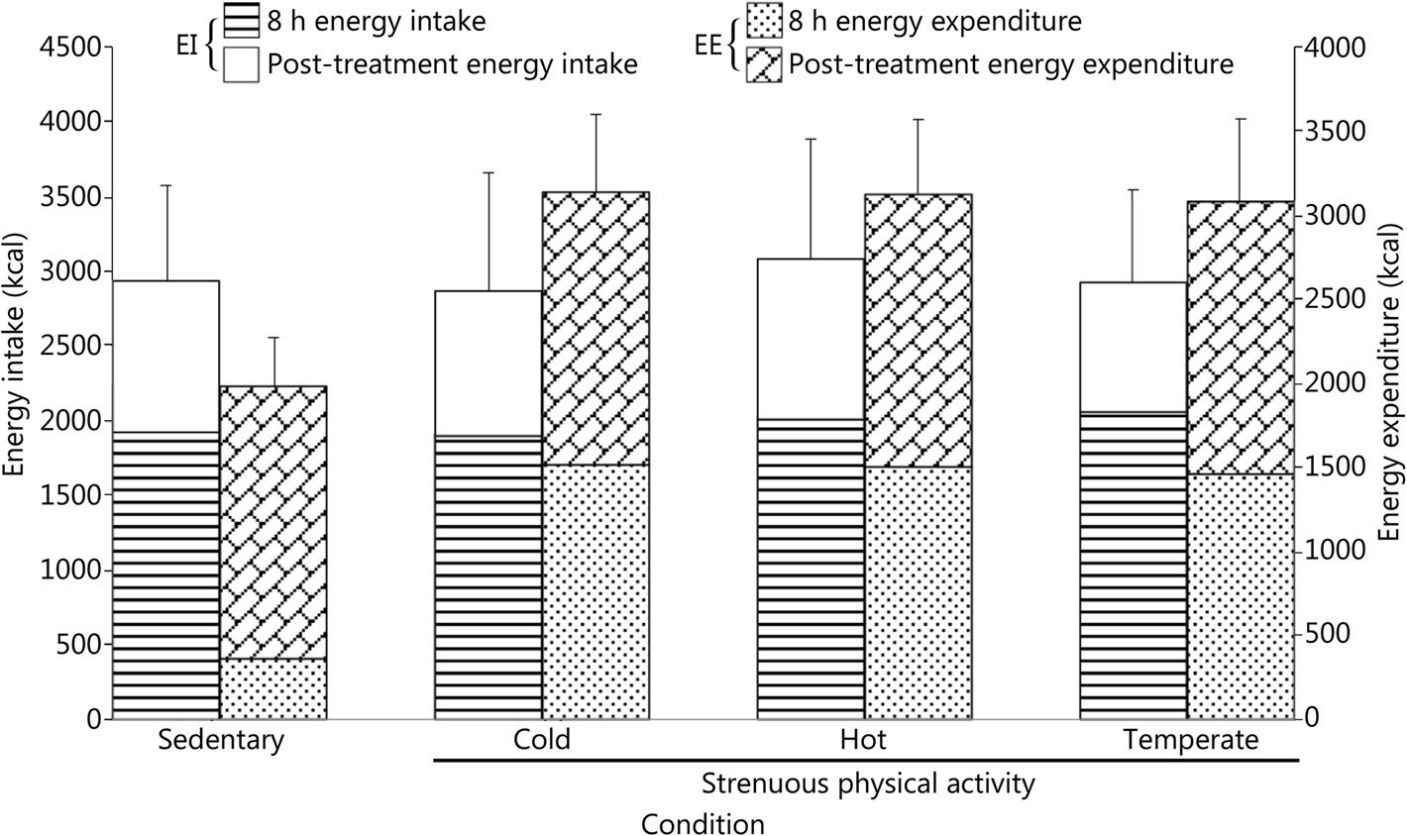
Comparison of energy intake with energy expenditure during the 8 h experimental treatment and during the 6 h posttreatment period (as part of a randomized experimental trial). Study participants were Regular Force or Class A Reservists (*n =* 18). The data presented are for 8 h and 16 h (a total of 24 h) of energy intake and expenditures (39) of CAF personnel participating in the four experimental treatments (sedentary (21 °C), cold (− 10 °C), hot (30 °C) and temperate (21 °C); humidity (~ 30% under sedentary, hot and temperate treatments and 50% in the cold treatment). Energy intake data were collected using the measured food intake/food waste method, where all consumed and/or non/partially consumed food and beverage were weighed and recorded for each participant. Strenuous physical activity consisted of infantry activities during the 8 h experimental treatments (for detailed analyses on energy expenditure, see Mandic et al. [40]).

There were no differences in energy intake between the experimental treatments during the 8 h when the participants were in the environmentally controlled chamber or during the remaining hours for the at-home posttreatments (Table 3, Fig. 3), although total energy expenditure was higher in the experimental treatment with strenuous physical activity in comparison with the energy expenditure associated with the sedentary treatment (for both 8 h and 24 h) but was not different between the 3 temperature conditions (Fig. 3) (for detailed analyses on energy expenditure, see Mandic et al. [40]).

### Urinary and blood biomarkers

Although there were no significant differences between experimental treatments in urinary creatinine, potassium or cortisol (*P* > 0.05), urinary urea associated with the hot treatment was significantly lower than that associated with the sedentary treatment (*P* ≤ 0.05), but the hot treatment urinary urea was not different than that associated with the cold or temperate treatments (*P* > 0.05, Table 4). Urinary sodium was significantly lower in the hot treatment (97 mmol/d) than in both the sedentary (156 mmol/d) and cold treatments (146 mmol/d, *P* ≤ 0.05), but it was not different from that in the temperate treatment (111 mmol/d, *P* > 0.05).

**Table 4:** Comparison of the effects of the different treatments on urinary and blood biomarkers in CAF personnel. [*Mean ± SD*]

Biomarker^1^	Sedentary	Strenuous physical activity^2^			*P*-value
		Cold	Hot	Temperate	
Urinary creatinine (mmol/d)	16 ± 4	15 ± 4	15 ± 5	15 ± 6	0.68
Urinary urea (mmol/d)	406 ± 92^a^	376 ± 113^ab^	327 ± 83^b^	359 ± 115^ab^	0.03*
Urinary sodium (mmol/d)	156 ± 56^a^	146 ± 68^a^	97 ± 52^b^	111 ± 68^b^	0.001*
Urinary potassium (mmol/d)	56 ± 16	50 ± 17	48 ± 16	45 ± 19	0.24
Urinary cortisol (nmol/L)	86 ± 43	73 ± 20	90 ± 59	90 ± 54	0.57
Urea (mmol/L)	5 ± 0.8	5 ± 1.1	5 ± 1.3	5 ± 0.9	0.17
Creatinine (μmol/L)	78 ± 12	79 ± 14	80 ± 15	79 ± 15	0.26
eGFR^1^ [ml/(min·1.73 m^2^)]	96 ± 9	95 ± 11	92 ± 13	95 ± 12	0.15
Triglycerides (mmol/L)	1.1 ± 0.7	0.9 ± 0.3	0.9 ± 0.3	1 ± 0.6	0.38
Cholesterol (mmol/L)	4.3 ± 0.8	4.1 ± 0.7	4.3 ± 0.8	4.1 ± 0.7	0.19
HDL cholesterol (mmol/L)	1.3 ± 0.5	1.3 ± 0.4	1.3 ± 0.4	1.3 ± 0.4	0.40
Cholesterol/HDL ratio (mmol/L)	3.5 ± 0.9	3.3 ± 0.9	3.4 ± 1.0	3.4 ± 1.1	0.38
LDL cholesterol (mmol/L)	2.4 ± 0.5	2.4 ± 0.6	2.6 ± 0.7	2.3 ± 0.6	0.18
Non-HDL cholesterol (mmol/L)	2.9 ± 0.6	2.8 ± 0.7	2.9 ± 0.8	2.8 ± 0.8	0.17
Ascorbic acid (μmol/L)	43 ± 16	42 ± 13	44 ± 14	39 ± 12	0.55

Study participants were Regular Force or Class A Reservists (*n =* 18). The data presented are for 24 h urinary biomarkers and blood biomarkers (collected the day after the experimental trial visits [sedentary (21 °C), cold (− 10 °C), hot (30 °C) and temperate (21 °C); humidity (~ 30–50%)] of CAF participants. ^1^Urinary and blood biomarker data were examined by Linear Mixed Models with Bonferroni adjustment. ^2^Strenuous physical activity consisted of infantry activities during the 8 h experimental treatments. Data are presented as the means ± standard deviations (SDs). ^a,b^Values with different superscripts indicate statistically significant differences (*P* ≤ 0.05); ^*^P-value is the significance level for differences between the experimental treatments. ^1^Calculated eGFR as provided by Lifelabs and calculated as 186 × (Creatinine/88.4)^-1.154^ × (Age)^-0.203^ × (0.742 if female) × (1.210 if black)

## Discussion

The primary aim of this study was to investigate the acute impact of environmental stress along with strenuous physical activity on energy and nutrient intakes from field rations in a convenience sample of CAF personnel consuming field rations *Ad libitum*. As reported by others [4, 12, 29], only a portion of the ration meals (~ 70% of the amount provided) was consumed even in trials where energy expenditure was markedly elevated, and even though participants had ample time to eat and the meals were prepared on request. Even with an acute challenge of harsh environmental temperatures and strenuous physical activity, there were no differences in energy intake between the experimental treatments. As a result, energy intake did not increase in relation to increased energy expenditure under conditions of temperature stress with strenuous physical activity in comparison to the energy intake in the resting thermoneutral environment (sedentary, 21 °C), which was indicative of potential voluntary anorexia. Furthermore, as demonstrated by the total amount of energy consumed in the postexperimental treatments from the dinner ration packs, participants did not compensate for increased energy expenditure during the experimental treatments by increasing their energy intake when they had ample time to eat and the conditions of stress (e.g., strenuous physical activity) had subsided. Our findings also indicated that the palatability of the rations was not a factor in the consumption of the field rations (Additional file 1: Table S1). As a result, participants in this study are likely at an increased risk of voluntary anorexia, thereby impacting operational readiness and performance if the experimental treatments were to be conducted for a longer period of time [31, 33] than investigated in this study.

The environmental temperatures sustained by participants in the four experimental treatments were similar to those reported in previous studies. For example, Tharion et al. [11] investigated the energy requirements of US Marines in hot climate conditions where the temperatures ranged from 23.4–29.8 °C, while in a study by Consolazio et al. [46], the temperatures ranged from 30 to 34 °C, which was similar to the hot trial temperature (30 °C). Similarly, the temperatures in the temperate trial (21 °C) were comparable to those reported in previous studies (range 10–25 °C) [23, 47, 48]. The cold temperature in this study (− 10 °C) was also similar to the temperatures encountered by military personnel in several cold field trials that have previously been conducted [15, 16], although the temperature was different (either higher or lower) than that used in some other studies [12, 49, 50] (range 9 °C to <− 42 °C).

As expected and as reported in the companion manuscript to the current study [40], the energy expenditure as a result of the simulated military tasks performed by participants during the harsh environmental temperatures (~ 1700 kcal/4 h) was 76% of the energy expenditure during the sedentary experimental trial (404 kcal/4 h). To our knowledge, the participants in this study underwent the longest duration of strenuous physical activity compared to the durations used in previous studies of laboratory assessments of temperature extremes on energy intake [21, 22, 36] (≤2 h). However, the estimated energy expenditure for 24 h in this study (~ 3500 kcal/24 h) [40] was at the lower end of the energy expenditure found in other field trials (ranging from 3300 to > 6000 kcal/d) [12, 49]. However, it is likely that our 24 h estimates of energy expenditure may underestimate the true energy expended because they were not measured beyond the experimental trial session [40]. Our results of insufficient energy intake relative to energy expenditures under conditions of strenuous physical activity in ambient temperature extremes (hot/cold) are comparable to those of other studies, where other researchers have demonstrated insufficient energy intake (45 to 99% of energy expended, depending on duties/conditions/activities) relative to energy requirements as a result of both strenuous physical activities and temperature stress [4, 12, 13, 16, 33, 48, 49, 51]. This has important implications for energy deficits, weight loss and performance if the study were to be conducted in these temperature extremes (hot/cold) for longer periods of time than was examined in this investigation. Even in the face of these very significant intertrial differences in energy expenditure, energy intakes (as presented in this manuscript) were virtually identical for the experimental treatments with strenuous physical activity and for the sedentary treatment, averaging approximately 1970 kcal/8 h. Thus, our results show that during the acute period of this heightened energy expenditure, the participants incur an energy deficit regardless of the ambient temperature and food availability.

The results of the food satisfaction surveys (Additional file 1: Table S1) indicate that participants generally had high acceptability of field rations in the following categories of satisfaction (taste, texture, variety, quantity, saltiness, sweetness, density/fullness, digestibility and overall adequacy); therefore, it is unlikely that the palatability of the selected field rations would have impacted the consumption of rations during or after the trial, as suggested by others [52, 53]. Additionally, our previous results have shown that participants’ usual at home energy intake prior to the four experimental treatments did not differ from the energy intake measured on treatment days, suggesting that participants do not increase their intakes in relation to energy expenditure or eating environment (at home vs. in the lab) [54]. The findings did not change as a result of adjusting for any of the demographic characteristics of the participants (age, sex, BMI and percent body fat).

Our measured food intake/waste from field rations for the complete 24 h demonstrated that the participants consumed similar amounts of field rations posttreatment and did not compensate for their increased energy expenditures during the strenuous physical activity in harsh temperatures. It could be possible, however, that the compensatory appetite response to an acute high energy expenditure period is not matched by immediate energy intake but is delayed and may evolve over longer periods (i.e., in multiday operational settings) [55], which was not measured in this study. This is relevant since most military deployments (whether training or operational) consist of multiday or prolonged multi-week periods where high energy expenditure is experienced in austere environments and where field feeding is conducted in suboptimal conditions [3, 4, 56].

Although our study was not designed to evaluate the different requirements for macronutrients under temperature extremes, we did not observe any differences in macronutrient preference or intake between the different treatments, which is consistent with previous research [14, 15, 57]. Although participants in this study consumed only ~ 70% (averaged for all experimental treatments) of the total carbohydrates provided in the ration packs during the 8 h, the energy contribution from carbohydrates was within the AMDR (45–65%), although at the top of this range (64%). Carbohydrate intake from 24 h of consuming field rations was 5.6 g/(kg·d) (295 kcal/d, averaged for four treatments), which was higher than that demonstrated in other studies [3.2 g/(kg·d)] [12, 16] due to the high carbohydrate levels found in the field rations.

Several concerns have been raised regarding the consumption of higher fat diets in military personnel (as a result of consuming nonration foods such as high-fat animal foods found in countries with cold climates) and its association with increased cardiovascular risk [57, 58]; however, the fat content of the ration packs was generally low and was well-within the acceptable AMDR. In this study, fat intake was 1.16 g/(kg·d) (92 g/d, averaged for the four treatments), which was comparable to other studies in which intakes were 1.2 g/(kg·d) [11, 12]. In contrast, saturated fat intake accounted for 10% of the total energy intake (averaged across the four experimental conditions for 24 h), which was at the recommended limit of < 10% of total calories for military personnel (Military Dietary Reference Intakes) [59].

Findings from the literature suggest that increased protein intake is likely beneficial for maintaining muscle mass, strength and performance during strenuous physical activity and under conditions of negative energy balance [60–62]. The protein intake of 1.25 g/(kg·d) (99 g/d, averaged for four treatments) found in this study is comparable to that seen in other studies [1.3 g/(kg·d)] [12]. Based on the level of protein intake for military personnel [1.61 g/(kg·d)] found to be protective for lean mass retention in relation to strenuous physical activity [60], participants were consuming less than the recommended levels of protein from the field rations during the 24 h study period but would meet these protein requirements if most or all of the field ration pack was consumed [30].

Although the intakes of carbohydrates, fat and protein were generally similar to those of previous studies conducted with military personnel consuming field rations [11, 12], these results were different from those of other studies [12, 13, 15, 16]. Factors such as the type/quantity of ration consumed, level and duration of physical activity per type/trade of work, clothing and participant demographics (e.g., body weight, which was slightly lower at 79 kg in our study compared to that of Johnson et al., (87 kg) [12]) could account for differences in macronutrient intakes.

We did not find differences in fiber intake between the experimental treatments; however, fiber intake in all treatments was lower than the recommended amount of 38 g (dietary recommended adequate intake of fiber for males in Canada) [45]. Since field rations contain, on average, 31 g of fiber, likely as a result of the low content of fresh fruits and vegetables or high fiber foods, it may be difficult to obtain the recommended intake of fiber without consuming the complete ration pack with additional supplementation.

We did not observe significant differences in the intakes of several micronutrients when comparing the different treatments. Research indicates that there might be altered micronutrient requirements under temperature extremes as a result of impaired intestinal absorption or increased utilization for maintaining thermoregulation [63–66]. Additionally, recent studies have indicated the importance of nutrients such as iron, calcium, vitamin D and folate in sustaining performance and preventing injury, especially for female military personnel [64, 67]. Although short-term field operations are unlikely to lead to micronutrient deficiencies unless there is a pre-existing depletion of stores, lower than recommended intakes of micronutrients may have impacts on both physical and cognitive performance in a longer-term setting [64, 66]. As such, it is important to ensure that recommended amounts of micronutrients are provided for military personnel operating in climatic extremes, especially in view of voluntary anorexia.

Vitamin C intake was relatively high in the temperate and hot treatment groups in comparison to the intake in the cold and sedentary treatments. However, we did not find an acute impact of experimental temperature extremes on intake or plasma ascorbic acid levels (a nutritional biomarker for vitamin C status). In the absence of fresh fruits and vegetables in the field rations, participants were obtaining most of this nutrient in the temperate and hot treatment groups through the vitamin C-fortified drink crystals (as part of the field ration packs) mixed with water. In view of the lack of compelling evidence to suggest that experimental temperatures may have an impact on vitamin C intake or status, maintaining recommended levels of vitamin C intake predeployment and during deployment is likely sufficient to ensure nutrient adequacy in military personnel operating in temperature extremes, at least in an acute setting.

Our results demonstrated that CAF participants consumed higher than recommended amounts of sodium, even with the partial consumption of ration packs. There has been significant emphasis on reducing the sodium content of Canadian foods and the sodium intake of the Canadian population [68]; however, sodium intake recommendations do not take into account the increased amounts of sodium lost from skin through sweat in military personnel operating in temperature extremes [69]. Although we did not quantify the sodium lost in sweat, we observed significantly lower urinary sodium excretion in the hot environment than in the sedentary and cold treatments, which is likely attributed to hyponatremia, a consequence of fluid ingestion and increased sweat production [19, 70, 71]. This finding may have implications for military personnel being able to tolerate extra sodium under situations of strenuous physical activity in climatic extremes [69], but tolerability may differ upon acclimatization to the environmental extremes [72]. Suggestions for preventing hyponatremia or dehydration for military personnel include monitoring hydration and urine and ensuring proper nutrition (e.g., consideration of electrolyte-rich beverages) and rations according to conditions and activities [73]. Recently, a water planning algorithm has been developed that accurately predicts the water needs of military personnel across a wide range of environmental conditions, thus helping to minimize the risk of hyponatremia or dehydration [74].

It is well established that adequate hydration levels have significant impacts on the health and performance of military personnel; therefore, maintaining adequate intakes of water is crucial during operations in temperature extremes that include strenuous physical activity. The current US military fluid replacement guidelines, which are also applicable to the CAF hydration recommendations, suggest 1.5 L/h as an upper limit or 12 L of daily water intake in extreme hot environmental temperature conditions [75, 76], while water requirements in cold environments range from 2 L to 6 L daily, depending on the intensity and duration of physical activity [77]. In this study, participants consumed significantly less water in the cold treatment than in the hot and temperate environments, although the water intake in the hot/temperate conditions was less than the recommended intake (~ 4 L for 4 h of activity vs. the recommended ~ 6 L for the same hours of activity). This finding may have implications for dehydration in the context of environmental extremes (hot or cold) as a result of increases in fluid loss by sweating through heavy clothing or respiratory water losses in combination with increased metabolic activity [57, 78, 79]. In contrast to other studies that have indicated a strong correlation between water and food consumption [50, 80], the results from this study showed a significant difference in water intake between experimental treatments even though energy intake did not differ. Factors other than the availability of food are likely to influence water consumption while operating in climatic extremes. These factors may include decreased thirst sensation, activity level or the respiratory losses of water under environmental stress [17, 57, 78].

The strengths of this study include the use of measured food intake/waste to assess dietary intake during the experimental treatments and at home after study completion. In addition, this study benefited from high compliance of the study participants and minimal misreporting bias [81], likely due to the one-on-one training of each participant to ensure accurate recording of dietary intakes during the postexperimental treatment period, which may not be feasible for larger studies. Additionally, because rations are standardized compared to usual diets, variations in the nutritional composition of foods were also minimized. There are also some limitations in this study. The nutrition information for the field rations was obtained from the CAF Directorate of Food Services based on manufacturer-supplied Nutrition Facts tables or chemical analyses, except for the contents of some supplemented vitamins and minerals (B vitamins, zinc, phosphorus and magnesium), which were obtained from the Canadian Nutrient File 2013 (the online national database of the nutrition composition of Canadian foods, maintained by Health Canada) [42], using values from comparable canned, packaged or dehydrated foods. However, these values may not accurately estimate the actual nutrient levels in field rations. In dietary studies, participants may not enter all of the food items consumed and may not be able to provide accurate assessments of portion sizes [41]; however, these limitations of traditional dietary assessments are unlikely here because the ration packages were all preweighed and all uneaten foods were weighed.

This study is limited by a small sample size and was not designed to assess the impact of prolonged strenuous physical activity in temperature extremes on nutrient intakes. Thus, these results likely underestimate the implications of such energy deficits for military personnel engaged in more prolonged field operations under metabolically challenging operations [31, 33]. Additionally, this study is limited by a small sample of females; therefore, there was not enough power to analyze their data separately.

## Conclusions

This study demonstrated that, in comparison with energy consumed during the sedentary treatment, military personnel had similar energy and macronutrient intakes from field rations, even with an acute challenge of increased physical activity and temperature stress in this experimental trial designed to simulate field conditions (hot, cold and temperate) with strenuous physical activity. This study shows that, even when participants had ample time to consume the food and the food was prepared on request, participants did not consume sufficient rations to offset the increase in energy expenditure. As a result, there was a gap between the amount of food provided versus the amount that was consumed, which would be further compounded in the field due to conditions such as load carriage, cooking and prolonged exposure to temperature extremes and arduous physical demands. Additionally, this study also demonstrated that military personnel did not compensate for the reduced energy intake during the experimental treatments, even when they had time to eat and the stresses of strenuous physical activities in harsh environmental temperature had subsided (i.e., postexperimental treatment at home).

## Additional file

Additional file 1: **Table S1.** Responses to the food satisfaction survey (DOCX 17 kb)

## Data Availability

The datasets used and/or analyzed during the current study are not publicly available due to the wording in our informed consent forms that allowed participants to opt-out of consenting to the secondary use of their data. Data from participants who consented to the secondary use of their data will be made available by the corresponding author on reasonable request.

## Abbreviations

AMDR: Average macronutrient distribution ranges
BMI: Body mass index
CAF: Canadian armed forces
DRDC: Defence Research and Development Canada
DRI: Dietary reference intakes
IAs: Incremental allowances
PAR-Q^+^: Physical activity readiness questionnaire
