# Supplementary material for: Comparison of dietary intakes of Canadian Armed Forces personnel consuming field rations in acute hot, cold, and temperate conditions with standardized infantry activities
Source: Mil Med Res. 2019 Aug 16;6:26. doi: 10.1186/s40779-019-0216-7 (PMC6696676; doi:10.1186/s40779-019-0216-7)
Supplement: Supplementary file 1 — Table S1. Responses to the food satisfaction survey (DOCX 17 kb) [file 40779_2019_216_MOESM1_ESM.docx]

# Additional files

## Food satisfaction surveys

**By meal type (by breakfast, lunch and dinner):**

Participants (*n=*18) were provided with a food satisfaction survey for every meal (standardized for Canadian field rations and used in previous studies) to complete during the experimental trials and post-experimental trial. The food satisfaction survey had 13 questions with responses provided on a 5-point Likert-scale. The supplement tables show responses for 9 of those questions as the other 4 questions were on ease of preparation/consumption, preparation time, and food temperature that were not applicable during the experimental trials as the food was prepared by study coordinators.

A Kruskal-Wallis H test showed that there was no significant difference in variety, quantity, taste, texture, variety, quantity, saltiness, sweetness, density/fullness, digestibility and overall adequacy between the different meal types, (*χ*^2^ =2.3, 0.1, 0.03, 0.06, 0.08, 0.08, 0.05, 0.6, 0.01), *P*>0.05 (See supplementary table).

Overall, the menu items within the breakfast, lunch and dinner were scored largely acceptable in the different categories (taste, texture, variety, quantity, saltiness, sweetness, density/fullness, digestibility and overall adequacy) with a few at borderline acceptability.

**Additional file 1: Table S1** Responses to the food satisfaction survey

| Meal Type | Minimum | 25th | 50th | 75th | Maximum |
| --- | --- | --- | --- | --- | --- |
| Breakfast |  |  |  |  |  |
| Variety | 2 | 3 | 4 | 5 | 5 |
| Quantity | 3 | 3 | 4 | 5 | 5 |
| Taste | 2 | 3 | 4 | 4 | 5 |
| Texture | 2 | 3 | 4 | 4 | 5 |
| Salitness | 2 | 3 | 4 | 4 | 5 |
| Sweetness | 2 | 3 | 4 | 4 | 5 |
| Density and fullness | 2 | 3 | 4 | 4 | 5 |
| Digestibility | 2 | 3 | 4 | 4 | 5 |
| Overall adequacy | 1 | 2 | 4 | 4 | 5 |
| Lunch |  |  |  |  |  |
| Variety | 2 | 3 | 4 | 5 | 5 |
| Quantity | 3 | 4 | 4 | 5 | 5 |
| Taste | 1 | 2 | 4 | 5 | 5 |
| Texture | 2 | 3 | 4 | 4 | 5 |
| Salitness | 2 | 2.5 | 4 | 4 | 5 |
| Sweetness | 2 | 3 | 4 | 4 | 5 |
| Density and fullness | 2 | 3 | 4 | 4 | 5 |
| Digestibility | 2 | 3 | 4 | 5 | 5 |
| Overall adequacy | 2 | 3 | 4 | 4 | 5 |
| Dinner |  |  |  |  |  |
| Variety | 2 | 3 | 4 | 5 | 5 |
| Quantity | 3 | 4 | 4 | 5 | 5 |
| Taste | 2 | 3 | 4 | 5 | 5 |
| Texture | 2 | 3 | 4 | 5 | 5 |
| Salitness | 2 | 3 | 4 | 4 | 5 |
| Sweetness | 2 | 3 | 3 | 5 | 5 |
| Density and fullness | 2 | 3 | 4 | 5 | 5 |
| Digestibility | 2 | 3 | 4 | 5 | 5 |
| Overall adequacy | 2 | 3 | 4 | 4 | 5 |

The food satisfaction surveys asked participants to respond on a 5-point Likert scale. The numbers in the tables 1-3 indicate the minimum, maximum and percentiles (25th, 50th and 75th) of the Likert-scale responses by participants.
